# A233 PHENOTYPE OF MUSCULOSKELETAL MANIFESTATIONS IN A CANADIAN INCEPTION COHORT OF PEDIATRIC PATIENTS WITH INFLAMMATORY BOWEL DISEASE

**DOI:** 10.1093/jcag/gwad061.233

**Published:** 2024-02-14

**Authors:** E Dzongowski, N Suthar, T Walters, A Griffiths, W El-Matary, E I Benchimol, J deBruyn, R Berard, E Crowley

**Affiliations:** Pediatrics, Western University Schulich School of Medicine & Dentistry, London, ON, Canada; London Health Sciences Centre Children's Hospital, London, ON, Canada; SickKids Research Institute, Toronto, ON, Canada; SickKids Research Institute, Toronto, ON, Canada; University of Manitoba Max Rady College of Medicine, Winnipeg, MB, Canada; SickKids Research Institute, Toronto, ON, Canada; University of Calgary Cumming School of Medicine, Calgary, AB, Canada; London Health Sciences Centre Children's Hospital, London, ON, Canada; London Health Sciences Centre Children's Hospital, London, ON, Canada

## Abstract

**Background:**

Musculoskeletal (MSK) manifestations, including arthritis and arthralgia, are among the most common extraintestinal manifestations (EIMs) of inflammatory bowel disease (IBD), reported in 20-30% of adult patients [1]. However, there is a paucity of data regarding MSK EIMs in the pediatric IBD population. A recent systematic review [2] found only 13 studies with limited results, due to heterogenous study design and data reporting methods. As such, little is known regarding frequency of MSK EIMs, their phenotype, or factors associated with their development.

**Aims:**

The purpose of this study was to determine the frequency of MSK EIMs in a contemporary cohort of Canadian pIBD patients and describe the phenotype of MSK EIMs in this population.

**Methods:**

This was a prospective longitudinal cohort study with data from the inception cohort of CIDsCaNN (Canadian Children IBD Network) comprising patients aged 2-17 from 12 Canadian pediatric centres, from 2014 to 2021.

MSK EIM frequency was calculated for the cohort, and for subgroups by age, sex, and IBD type. MSK-EIM phenotype was described from case report forms with specific MSK features reported. Variables were compared with Pearson Chi-square or Fisher’s exact test. For patients without MSK EIM at IBD diagnosis, a Cox regression survival analysis was used for time to MSK development, with right censoring for patients who never reported MSK EIMs.

**Results:**

1,330 pIBD patients were included, with 761 males (57.2%) and 569 females (42.8%). There were 824 CD (62.0%), 382 UC (28.7%), and 124 IBD-U patients (9.3%).

81 patients (6.1%) reported MSK EIMs, with 63 CD (7.6%), 10 UC (2.6%), and 8 IBD-U (6.4%). CD patients were more likely to have MSK EIMs (7.6% vs 3.6% UC/IBD-U, p=0.002). 47 patients (58.0%) had MSK EIMs at or before diagnosis while 34 had them ampersand:003E4 weeks after diagnosis. There was no difference in overall frequency between sex or age groups, or time to MSK EIM development by age or ethnicity. Females were more likely to develop MSK EIMs, with odds increasing by 4.68 for each year after diagnosis (p=0.047).

59 (74.7%) MSK patients were seen by a rheumatologist. Peripheral MSK disease was in 51 patients (63%), axial disease only in 37. Peripheral and axial MSK symptoms followed the course of bowel disease in 40.3% and 28.1% of patients respectively, which were not significantly different.

**Conclusions:**

MSK EIMs affect 6.1% of a contemporary cohort of Canadian pIBD patients.This is less than reported in literature, likely due to physician-reported nature of our data. Our next step is to compare pIBD patients with MSK EIMs to a matched group without, for bowel disease-related outcomes and response to medications.

**Funding Agencies:**

Cassie and Friends Care and Research Network (CREW) funding.

